# The influence of heavy metals on biological soil quality assessments in the *Vaccinium myrtillus* L. rhizosphere under different field conditions

**DOI:** 10.1007/s10646-021-02345-1

**Published:** 2021-01-26

**Authors:** Marta Kandziora-Ciupa, Aleksandra Nadgórska-Socha, Gabriela Barczyk

**Affiliations:** Faculty of Natural Sciences, Ecology, Institute of Biology, Biotechnology and Environmental Protection, University of Silesia in Katowice, Bankowa 9, 40-007 Katowice, Poland

**Keywords:** Bilberry, Forest soils, Soil pollution, Soil quality, Root zone

## Abstract

The aim of this study was to determine the influence of heavy metals on biological soil quality assessments in *Vaccinium myrtillus* L. rhizosphere soil as well as in non-rhizosphere soil from different polluted sites. The presented study was also conducted in order to determine any differences in the soil physicochemical and biological properties between the *Vaccinium* rhizosphere soil and the non-rhizosphere soil. The content of heavy metals and their potential bioavailability, content of macronutrients, physicochemical soil properties, activity of six soil enzymes and microarthropod communities were determined. Soil organic matter, the levels of *C*, *N* and all the studied macronutrients and almost all enzyme activity were significantly higher in the rhizosphere soil than in the non-rhizosphere soil. At the most contaminated site, the content of heavy metals was also higher in the rhizosphere soil, but their bioavailability was lower than in the non-rhizosphere soil. The β-glucosidase and urease activity in the soil correlated most negatively with the examined metals. The levels of two enzymes were also strongly impacted by the organic matter—the *C* and *N* levels and pH. The number of microarthropods as well as the QBS (soil biological quality index) and *F*_EMI_ (abundance-based fauna index) were higher in the rhizosphere soil. The bilberry rhizosphere soil had stronger correlation coefficient values between the measured parameters than the non-rhizosphere soil, which suggests that rhizosphere soil is more sensitive and could be used in the monitoring and assessment of forest ecosystems. β-glucosidase and urease were the most sensitive indicators of the adverse impact of Cd, Zn and Pb. The *F*_EMI_ index seems to be a better indicator than the QBS for identifying differences in soil quality.

## Introduction

Soils are often a sink for pollutants especially for heavy metals in anthropogenic environments (Jiao et al. 2015; Navarrete et al. 2017). Heavy metals in soils, which are characterized by a high stability in the environment and are generally not biodegradable, can be released from terrestrial environments into other ecosystem compartments such as groundwater, rivers, atmosphere and other (Mmolawa et al. 2011; Mazurek et al. 2017). They can reach hazardous levels to human beings, hence, the need for constant monitoring and regulation of their concentrations in the soil (Karaca et al. 2010; Jia et al. 2018). Soil physicochemical properties are not suitable for estimating changes in environmental pollution because they change very slowly and can only be detected after many years. Therefore, any research on pollution-induced changes in soil quality must be based on the properties that respond rapidly to minor changes in environmental stress (Trasar-Cepeda et al. 2008; Tan et al. 2014). One of the suggested biological indicators is soil enzyme activity level, which rapidly responds to any ecosystem variation and changes in the soil, including those that are induced by heavy metals. Because it is easily measured, it could provide a useful tool for environmental monitoring (Rao et al. 2014). In addition, understanding the effects of heavy metals on soil enzyme activity may also provide an opportunity for an integrated assessment of soil biology (Yang et al. 2017). Increased contents of heavy metals in soil above a certain threshold generally adversely affect the growth, morphology, and metabolism of microorganisms, which leads to a decrease in the functional diversity of soil ecosystems (Hassan et al. 2013). They can inhibit soil enzyme activity by interacting with enzyme active sites and substrate complexes and denaturing the enzyme protein (Vig et al. 2003; Yang et al. 2017). Soil organisms are also useful in monitoring environmental changes because they provide objective metrics that integrate physical, chemical, and biological parameters (Blakely et al. 2002; Galli et al. 2014). For example, microarthropods are widely seen as bioindicators (Stork and Eggleton 1992; Paoletti 1999) and are used in the indexes of soil quality in environmental monitoring (Yan et al. 2011). Parissi et al. (2005) and Parisi and Menta (2008) proposed a simplified eco-morphological index (EMI) that is based on the types of soil microarthropods that are present and this index was used to evaluate soil quality by generating another index—the QBS (soil biological quality) (Yan et al. 2011). The QBS index is based on the concept that at a higher soil quality, the number of microarthropod groups that are well adapted to soil habitats will be higher (Parissi et al. 2005).

From the viewpoint of microbial ecology, the rhizosphere is a special unique hot spot in the soil where microorganisms are considerably stimulated by the activity of the roots (Jones et al. 2004; Hisinger et al. 2006; Egamberdieva et al. 2011). The rhizosphere is also an important site of material cycling and energy flow (Xiao et al. 2017) and has a significant influence on the availability or solubility of nutrients as well as on the availability of heavy metals (Orroño et al. 2012). Because of this, the distribution of heavy metals in rhizosphere soil is more significant for the evaluation of the bioavailability of heavy metals than bulk soil is (Youssef 1997).

*Vaccinium myrtillus* L. (bilberry) is the most frequent and abundant dwarf shrub species in the understory of the conifer forests in Europe and Northern Asia. As a species, it has special significance for the development of pine and mixed oak-pine forests undergrowth structure and is a species that is particularly important for the regeneration of post-agricultural forest communities. Bilberry makes a significant contribution to the soil processes in this community type because it is a major contributor to the formation and accumulation of the humus layer and in the prevention of soil erosion (Matuszkiewicz et al. 2013; Liu et al. 2014; Kandziora-Ciupa et al. 2017). However, information about the rhizosphere effect of *Vaccinium myrtillus* L. on soil properties, activities soil enzymes and microarthropod abundance, especially in heavy-metal contaminated soils, is still unknown. Therefore, the objectives of this study were to (1) evaluate the differences in the rhizosphere and non-rhizosphere soil properties from different contaminated stands; (2) assess the influence of heavy metal pollution on the enzyme activity in rhizosphere and non-rhizosphere soils; (3) compare the biological indicators of soil quality (QBS and *F*_EMI_) based on the microarthropod numbers in rhizosphere and non-rhizosphere soils. We postulate that the results of this study will contribute essential information for the monitoring and assessment of forests soils and will provide a better understanding of the processes that occur in rhizosphere soil that is under heavy metal contamination.

## Material and methods

### Study area

**Fig. 1:**
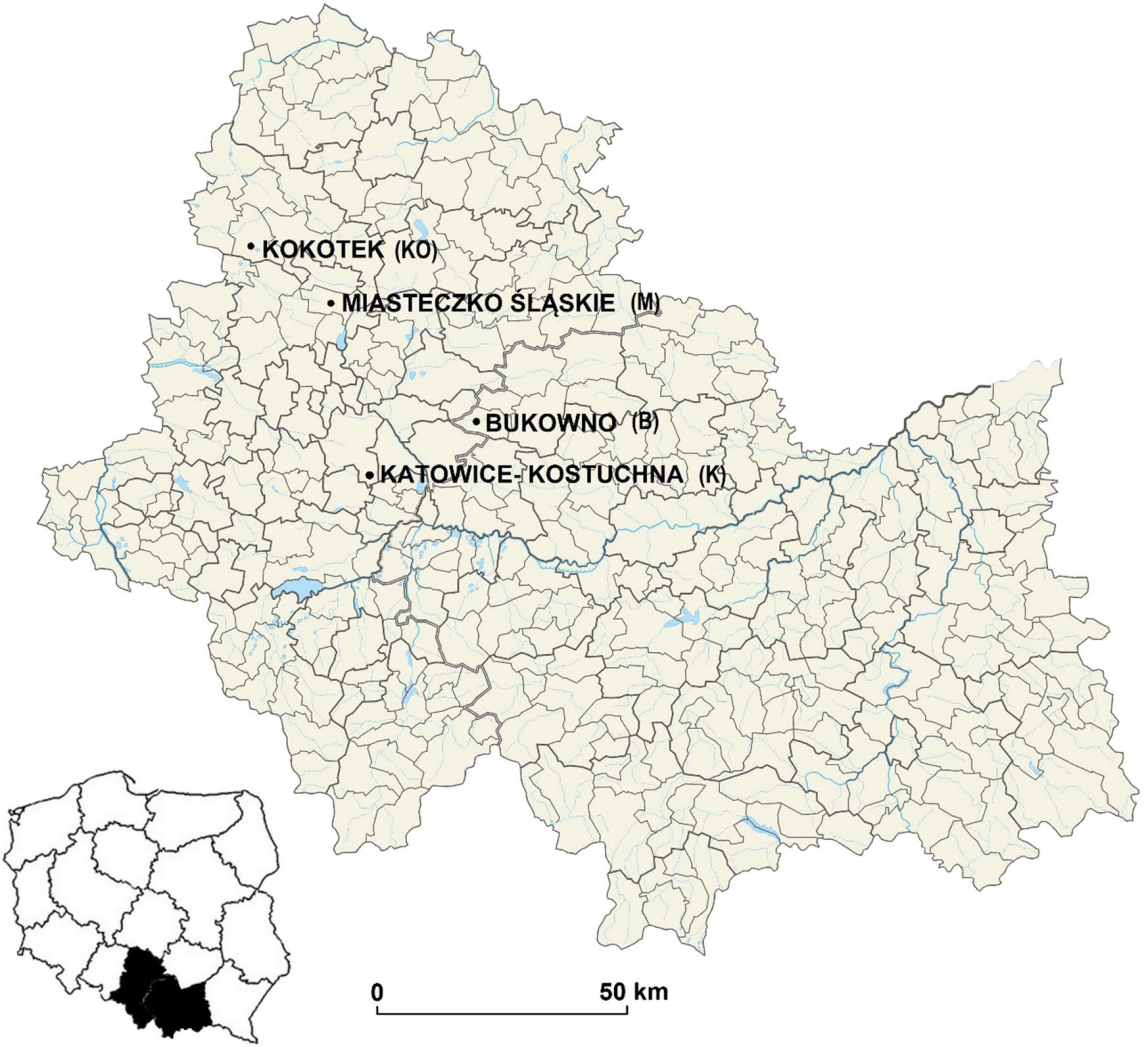
Location map of sampling sites

The study was performed in a middle-aged (60–80 years old) Scots pine forest, which is mixed with birch (*Betula pendula* L.), European beech (*Fagus sylvatica* L.) and pedunculate oak (*Quercus robur* L.) that are growing on sandy acidic soils that are located at three differently polluted sites (the immediate vicinity of the “Miasteczko Śląskie” zinc smelter (M), of the mining and metallurgical plant in Bukowno (B) and a main road with high traffic—Katowice–Kostuchna (K)) as well as in an unprotected natural forest community in Kokotek (KO) (Fig. 1). The dominant species in understory of all research areas was *Vaccinium myrtillus* L. (coverage in all sampling sites was 50–60%). All the sites are located in the Śląskie or Małopolskie provinces in southern Poland (in the Silesian-Krakow highlands) and all sampling sites were homogeneous in terms of altitude and exposure. The research areas are flat land located at an altitude of 200–314 m above sea level (Table 1).

**Table 1:** Study sites

Abbreviation	Sites	GPS	
		Latitude	Longitude
M	Nearest vicinity of zinc smelter “Miasteczko Śląskie” in Miasteczko Śląskie (activities since 1968)	50°31′22.655″N	18°56′8.699″E
B	The nearest vicinity of ZGH “Boleslaw” Mining and Metallurgical Plant in Bukowno (activities since 1955)	50°15′55.6″N	19°26′34.64″E
K	Katowice–Kostuchna province. vicinity of the main road. with high traffic	50°11′42.75″N	19°0′26.363″E
KO	Unprotected natural forest community in Kokotek—province of Lubliniec (control site)	50°36′21.287″N	18°42′59.806″E

### Sample collection

Soil samples were collected in May and September 2017 (Pennesi and Insom 2012). At each sampling site, ten randomly selected shrubs of *Vaccinium myrtillus* L. were carefully dug up from the field. The soil that was strongly adhering to the bilberry roots, which was separated by gently shaking by hand, was considered to be the rhizosphere soil (R) (Baudoin et al. 2002; Garcia et al. 2005; Ge et al. 2011). Ten samples were also collected from areas without vegetation or with light vegetation but without *V. myrtillus* (distances of at least 50 cm from the rhizosphere of individual plants in order to avoid the influence of rhizosphere)—for the sake of simplicity and for the needs of this article, we will call this soil “non-rhizosphere” soil (NR). At each study site, ten soil samples of each type of soil (separately rhizosphere and non-rhizosphere) that had been collected from all of the sampling sites were combined into three composite samples and then divided into two sub-samples: one subsample was used to determine the heavy metal content, the second was used for the physicochemical analysis and the third for the enzyme activity analysis. All the measurements were done in triplicate.

At each sampling site, soil samples were additionally collected for the study of the microarthropod community. A total of 48 soil samples (10 cm × 10 cm × 10 cm)—rhizosphere (24) and non-rhizosphere (24) was collected. The fauna was extracted over seven days using a Berlese–Tullgren funnel. The arthropods were preserved in 75% ethanol. The extracted specimens were counted under a stereo microscope at a low magnification and identified to the order level.

### Soil properties

Soil pH was measured using a 1:2.5 soil to water ratio. The organic matter content (%) was determined using the loss-on-ignition (LOI) method by heating 5.0 g of soil at 550 °C for seven hours following the method of Ostrowska et al. (1991).

The soil concentration of heavy metals (Cd, Mn, Zn, Fe, Pb) and macronutrients (K, Mg, Na, Ca, P, S) were estimated in air-dried soil samples that had been sieved through a 2 mm sieve according to Zheljazkov et al. (2008) and Wójcik et al. (2014). The metals and macronutrients were extracted from the samples with concentrated HNO_3_ (65%) (acid extracted elements) or with 0.01 M CaCl_2_ (potentially bioavailable elements—only metals). For the HNO_3_-extractable fraction, the soil samples (0.5 g) were placed in digestion tubes, soaked overnight in 5 ml of concentrated HNO_3_ at room temperature, then decomposed further on an aluminum digestion block at 150 °C for 8 h, filtered and diluted to 25 ml with deionized water. For the CaCl_2_ extraction, 5 g of soil with a 50 ml 0.01 M CaCl_2_ solution was mechanically shaken for 2 h at room temperature. The levels of the metals were measured in the filtered extracts using inductively coupled plasma-atomic emission spectroscopy (Spectro Analytical Instruments).

Total carbon, total nitrogen and the *C*/*N* ratio were measured in an Elementar Vario MAX CNS Analyzer.

### Assessment of heavy metal pollution

The single contamination factor (CF) was used to assess the degree of pollution for each investigated metal:

$${\mathrm{CF}} = {{C_s}}/{{C_c}}$$

where *C*_*s*_ (mg/kg) is the average concentration of elements in the samples and *C*_*c*_ (mg/kg) is the average concentration of the elements in the standards or control or an unpolluted area (Boamponsem et al. 2010; Yang et al. 2016; Fang et al. 2017). The contamination levels ranged from 1 to 6 (0 = none, 1 = none to moderate, 2 = moderate, 3 = moderate to strong, 4 = strongly polluted, 5 = strong to very strong and 6 = very strong) (Muller, 1969).

To calculate the overall level of soil pollution across the sampling sites, the pollution load index (PLI) was determined (Bhuiyan et al. 2010, Yang et al. 2016; Fang et al. 2017):

$${\mathrm{PLI}} = ({{C}}_{f1} \times {{C}}_{f2} \times {{C}}_{f3} \times \ldots . \times {{C}}_{fn})^{1/n}$$

where *C*_*f*_ is the metal contamination factor and *n* is the number of samples that were analyzed in this study. Four pollution levels were defined: no pollution (PLI < 1), moderate pollution (1 < PLI < 2), heavy pollution (2 < PLI < 3) and extremely heavy pollution (PLI > 3) (Liu et al. 2013; Yang et al. 2016; Fang et al. 2017). The PLI for each site’s overall pollution level was also calculated (Table 5).

### Soil enzyme activity

The activity of six enzymes (arylsulfatase (AS), β-glucosidase (βG), dehydrogenase (Deh), alkaline (AlP), acid (AP) phosphatase and urease (Ure)) were determined. Fresh soil samples (rhizosphere and non-rhizosphere) from all four sites were sieved through a 2-mm sieve and stored in plastic zip bags at 4 °C. The substrate, incubation time, unit, and references of all the enzyme activities that were measured are listed in Table 2.

**Table 2:** Methods of soil enzyme activity

Enzyme	Substrate	Incubation (h)	Unit	References
Arylsulphatase	Potassium 4-nitrophenyl sulfate	1	μg *p*-nitrophenol g^−1^ dm h^-1^	Tabatabai and Bremner (1970); Strobl et al. (1996)
β-glucosidase	Salicin	3	µg saligenin g^-1^ dm 3 h^−1^	Hoffamnn and Dedeken (1966); Strobl et al. (1996)
Dehydrogenase	Triphenyltetrazolium chloride (TTC)	16	μg TPF g^-1^ dm 16^−1^	Schinner et al. (1996)
Alkaline and Acid phosphatase	*p*-nitrophenolphosphate	1	µg *p*-nitrophenol g^−1^ dm h^−1^	Tabatabai and Bremner (1969); Schinner et al. (1996)
Urease	Urea	3	µg N g^−1^ dm 3 h^−1^	Schinner et al. (1996)

*dm* dry mass

To assess the total level of soil enzyme activity, the TEI (total enzyme activity index) was calculated:

$${\mathrm{TEI}} = {\sum} {\frac{{X_i}}{{X_\iota }}}$$

where *X*_*i*_ is the activity of soil enzyme *i* and $\overline {X_i}$ is the mean activity of enzyme *i* in all the samples (Tan et al. 2014; Fang et al. 2017).

The potential biochemical soil fertility index (*M*_*w*_), which is based on the enzymatic activity and carbon content (Wyszkowska and Wyszkowski 2003), were calculated as follows:

$${\mathrm{M}}_{\mathrm{w}} = ({\mathrm{Ure}}{\kern 1pt} 10^{ - 1} + {\mathrm{Deh}} + {\mathrm{AlP}} + {\mathrm{AP}}){\kern 1pt} \% {{C}}$$

where Ure is urease activity, Deh is dehydrogenase activity, AlP is alkaline phosphatase activity and AP is acid phosphatase activity.

To compare the effects of the heavy metals between the contaminated soils, the enzyme activity change ratio (ACR) was calculated (Xian et al. 2015; Gucwa-Przepióra et al. 2016):

$${\mathrm{ACR}} = \left( {{{A}}_{{h}}-{{A}}_{{c}}} \right)/{{A}}_{{c}} \times 100\%$$

where *A*_*h*_ and *A*_*c*_ are the enzyme activity of polluted (M, B and K) and control (KO) soils, respectively.

### Biological indices

The soil biological quality index (QBS) was evaluated as reported by Parissi et al. (2005). The QBS considers the soil microarthropods, which are invertebrates that belong to the microarthropoda phylum that range in size between 0.2 and 2 mm (mesofauna). This QBS index classifies soil microarthropods based on their morphological characteristics, assigning each to a microarthropod group by different weights, which are represented by a different score, thereby defining the Ecomorphological indices (EMI) and the microarthropod groups presented in Parissi et al. (2005). The QBS is calculated as the sum of the EMI values in each soil type (Menta et al. 2018).

Soil quality was also estimated using the abundance-based (number of individuals) fauna index (*F*_EMI_), which is based on the ecomorphological indices (EMI). This indicator, which was proposed by Yan et al. (2012), is based on the presence/absence of microarthropod groups and the abundance of individuals in those groups.

$$F_{EMI} = \frac{{S_0}}{S} \times \frac{{\mathop {\sum }\nolimits_{i = 1}^{S_0} \left( {\frac{{d_{i0}}}{{d_{imax}}} \times EMI_i} \right)}}{{\mathop {\sum }\nolimits_{i = 1}^S \left( {EMI_i} \right)}}$$

where *S* is the number of microarthropod groups at all of the sites in the study region *S*_0_ is the number of microarthropod groups at one site in the study regiond *i*_0_ is the abundance of microarthropod group *idi*_*max*_ is the maximum abundance of microarthropod group *i* in all of the sites in the study area and *EMI*_*i*_ is the ecomorphological index of microarthropod group *i*.

Keys used to the taxonomical identification: Identification key (a): https://www.zoology.ubc.ca/~srivast/mites/index.html; ver.1.0 Identification key (b): https://keyserver.lucidcentral.org/key-server/player.jsp?keyId=56 and Insom, La Terza (2012).

### Statistical analysis

The data concerning enzyme activity, metal content and other soil properties were checked for the normality and homogeneity of variance. When there was a normal distribution and variance homogeneity, the data was analyzed by ANOVA and the treatments were treated as the independent variables. Significant statistical differences of all of the variables were established using the Tukey’s test (ANOVA; Statistica 10 package). The heat maps, which are based on Pearson’s correlation coefficients and show the correlation between enzyme activity and soil characteristics, were generated using HEMI software (Heat Map Illustration, Version 1.0) (Deng et al. 2014). CANOCO 4.5 was used to perform the Principal Component Analysis, which assessed the similarities and relationships between the soil properties and soil enzyme activity in the studied areas. PCA analysis was performed without data rotation.

## Results

### Soil properties, macronutrient concentrations, heavy metal content and bioavailability in the soil samples

All of the investigated soils were acidic. The lowest soil pH value was observed at site KO while the highest was observed at site M. There was a no difference in pH between the rhizosphere and non-rhizosphere soils (Table 3). Statistically significant differences between the rhizosphere and non-rhizosphere soils were found in the amount of organic matter (OM), carbon and nitrogen content with higher contents recorded in the rhizosphere soil samples. The amount of organic matter ranged from 6.6% at site M (non-rhizosphere soil) to 68.6% at site KO (rhizosphere soil) (Table 3). The total concentrations of *C* and *N* and the *C*/*N* ratio differed slightly between the sampling sites (Table 3).

**Table 3:** The physicochemical properties of soil samples (mean values ± SD, *n* = 3)

			pH	OM %	*C* %	*N* %	*C*/*N*
M	V	R	5.4 ± 0.01a*	17.8 ± 2.0a*	9.58 ± 1.07a*	0.50 ± 0.05a*	19.26 ± 0.26a*
		NR	4.7 ± 0.05a^	11.4 ± 0.20a^	5.56 ± 0.20a^	0.26 ± 0.01a^	21.48 ± 0.09a^
	IX	R	5.1 ± 0.02a*	17.5 ± 0.90a*	8.16 ± 0.00a*	0.40 ± 0.01a*	20.35 ± 0.33a*
		NR	4.6 ± 0.01a^	6.6 ± 1.0a^	3.99 ± 0.42a^	0.19 ± 0.03^	20.94 ± 1.18a^
B	V	R	4.4 ± 0.02b*	49.4 ± 0.40c*	22.68 ± 0.54b*	0.80 ± 0.02b*	28.28 ± 0.05c*
		NR	5.0 ± 0.02b^	23.9 ± 0.90c^	9.62 ± 1.96b^	0.41 ± 0.08b^	23.35 ± 0.26b^
	IX	R	4.1 ± 0.01b*	64.7 ± 0.10b*	30.69 ± 0.09c*	1.10 ± 0.00b*	27.99 ± 0.03c*
		NR	5.3 ± 0.01b^	14.3 ± 0.10b^	5.64 ± 0.68b^	0.24 ± 0.03b^	23.20 ± 0.12a^
K	V	R	4.0 ± 0.01c*	38.9 ± 0.10b*	21.62 ± 1.23b*	0.92 ± 0.05b*	23.52 ± 0.06b*
		NR	4.2 ± 0.02c^	18.1 ± 0.50b^	9.85 ± 1.68b^	0.47 ± 0.06b^	21.16 ± 0.83a^
	IX	R	3.9 ± 0.01c*	47.6 ± 0.40c*	19.43 ± 1.19b*	0.85 ± 0.04c*	22.99 ± 0.21b*
		NR	3.8 ± 0.01c^	17 ± 0.00c^	8.19 ± 0.30c^	0.34 ± 0.01b^	23.97 ± 0.04a^
KO	V	R	3.5 ± 0.03d*	47.1 ± 1.90c*	24.77 ± 1.06c*	0.85 ± 0.06b*	29.24 ± 0.62c*
		NR	3.6 ± 0.03d^	26.9 ± 3.50c^	30.75 ± 0.06c^	1.17 ± 0.00c^	26.21 ± 0.09c^
	IX	R	3.6 ± 0.02d*	68.6 ± 0.40d*	19.97 ± 1.86b*	0.69 ± 0.05d*	28.83 ± 0.52c*
		NR	3.7 ± 0.01d^	8.8 ± 0.40d^	3.13 ± 0.01a^	0.10 ± 0.00a^	30.25 ± 1.03b^

The different letters denote significant differences between the particular soil physiochemical properties in the rhizosphere or non-rhizosphere soils in the same month and different marks denote significant differences between the rhizosphere and non-rhizosphere soils in the same month and in this same sapling sites (*p* < 0.05)

*M* Miasteczko Śląskie, *B* Bukowno, *K* Katowice–Kostuchna, *KO* Kokotek, *V* May, *IX* September, *R* rhizosphere soil, *NR* non-rhizosphere soil, *OM* organic matter

No significant differences were found in the K, Mg, Na, Ca, P and S levels between sampling sites. We observed statistically significant higher macronutrient concentrations (*p* < 0.05) in the rhizosphere soil than in the non-rhizosphere soil at all of the sampling sites (Table 4).

**Table 4:** The concentrations of macronutrients (mg kg^−1^) at soil samples (mean values ± SD, *n* = 3)

			K	Mg	Na	Ca	P	S
M	V	R	171.17 ± 23.19a*	285.70 ± 42.95c*	1.23 ± 0.18b*	690.75 ± 92.84ab*	818.22 ± 110.78a*	2028.68 ± 301.85a*
		NR	111.87 ± 4.67a^	126.72 ± 11.15a^	0.82 ± 0.03a^	368.68 ± 27.48a^	379.08 ± 25.59a^	938.72 ± 74.41a^
	IX	R	175.75 ± 4.94a*	218.50 ± 7.72b*	1.07 ± 0.03ab*	536.60 ± 23.99a*	653.15 ± 10.80a*	1695.25 ± 56.23a*
		NR	113.65 ± 12.76a^	96.70 ± 15.05a^	0.70 ± 0.05a^	107.92 ± 27.93a^	308.80 ± 40.76a^	746.73 ± 88.78b^
B	V	R	198.73 ± 26.26a*	210.38 ± 26.83bc*	1.10 ± 0.13a*	1415.88 ± 224.07c*	653.83 ± 70.72a*	1810.32 ± 234.59a*
		NR	186.10 ± 17.91b*	229.28 ± 27.73c*	1.60 ± 0.13c^	1085.57 ± 150.56c^	501.57 ± 66.73b*	1402.90 ± 171.19b*
	IX	R	207.55 ± 37.46a*	213.65 ± 39.19b*	1.12 ± 0.19b*	1522.97 ± 324.91c*	698.55 ± 120.91a*	1959.63 ± 368.36a*
		NR	161.15 ± 3.12c*	202.02 ± 5.23c*	1.45 ± 0.05c^	892.07 ± 49.08c^	354.60 ± 12.83ab^	1002.15 ± 42.85c^
K	V	R	184.23 ± 37.22a*	147.03 ± 41.18ab*	0.98 ± 0.19a*	795.73 ± 157.47b*	705.27 ± 158.08a*	1599.17 ± 352.92a*
		NR	175.65 ± 5.32b*	184.38 ± 5.13b*	1.15 ± 0.05b*	481.30 ± 32.04a^	593.90 ± 34.00b*	1272.33 ± 60.33b*
	IX	R	163.45 ± 6.49a*	143.22 ± 10.19a*	0.82 ± 0.03a*	738.97 ± 27.42a*	681.50 ± 28.98a*	1644.00 ± 63.68a*
		NR	137.00 ± 5.30b^	113.40 ± 8.25b^	0.83 ± 0.03b*	249.58 ± 28.94b^	425.42 ± 28.11b^	968.27 ± 80.36bc^
KO	V	R	137.73 ± 23.00a*	69.60 ± 19.72a*	0.73 ± 0.10a*	369.22 ± 76.62a*	680.98 ± 138.99a*	1452.90 ± 302.80a*
		NR	134.50 ± 2.98a*	89.75 ± 1.88a*	0.78 ± 0.03a*	279.00 ± 9.97a*	515.50 ± 16.46b*	1228.33 ± 18.13b*
	IX	R	182.10 ± 4.50a*	98.10 ± 9.09a*	1.02 ± 0.03ab*	816.37 ± 6.55a*	890.97 ± 12.76b*	1772.65 ± 16.45a*
		NR	99.73 ± 8.40a^	52.25 ± 10.71a	0.65 ± 0.05a^	71.55 ± 43.43a^	350.42 ± 60.29ab^	451.65 ± 113.58a^

The different letters denote significant differences between the particular macronutrient concentrations in the rhizosphere or non-rhizosphere soils in the same month and different marks denote significant differences between the rhizosphere and non-rhizosphere soils in the same month and in this same sapling sites (*p* < 0.05)

*M* Miasteczko Śląskie, *B* Bukowno, *K* Katowice–Kostuchna, *KO* Kokotek, *V* May, *IX* September, *R* rhizosphere soil, *NR* non-rhizosphere soil

There were statistically significant differences in the content of the studied metals (HNO_3_ extracted and CaCl_2_ extracted) between the polluted and control sites. Additionally, there was a clear difference in the concentrations of metals between the rhizosphere and non-rhizosphere soil samples (Tables 5 and 6).

**Table 5:** The concentration of selected metals (mg kg^−1^) in fractions of the soils extracted with HNO_3_ (mean values ± SD, *n* = 3) and classification of soil samples

			Cd	Mn	Zn	Fe	Pb	PLI	GRADE
M	V	R	33.53 ± 3.97b*	124.82 ± 16.46c*	1697.50 ± 221.96c*	4164.17 ± 582.73c*	1568.75 ± 343.65b*	22	EH
		NR	10.63 ± 0.76c^	52.02 ± 4.86bc^	640.70 ± 50.27b^	2451.48 ± 208.85a^	580.83 ± 15.73c^	9	EH
	IX	R	28.53 ± 0.76c*	79.43 ± 2.27c*	1407.08 ± 330.96b*	3965.83 ± 571.08c*	1584.58 ± 275.97b*	16	EH
		NR	6.00 ± 0.87b^	26.92 ± 3.04b^	319.83 ± 38.59b^	50411.25 ± 6541.68b^	657.92 ± 70.65c^	14	EH
B	V	R	3.07 ± 0.45a*	35.00 ± 2.36a*	456.95 ± 60.08b*	3813.33 ± 507.48b*	206.25 ± 24.33a*	5	EH
		NR	7.60 ± 1.00b^	64.78 ± 8.43c^	710.80 ± 104.83b^	4476.67 ± 585.65c*	379.58 ± 54.98b^	9	EH
	IX	R	2.95 ± 0.56b*	26.75 ± 4.54a*	461.27 ± 81.61a*	3272.08 ± 434.66bc*	203.33 ± 39.69a*	4	EH
		NR	5.50 ± 0.35ba^	45.32 ± 1.21c^	532.40 ± 23.48c*	3382.92 ± 180.11a*	287.50 ± 11.25b^	14	EH
K	V	R	0.40 ± 0.13a*	79.62 ± 4.65c*	40.42 ± 11.11a*	2196.47 ± 596.86a*	61.55 ± 17.02a*	2	M
		NR	0.30 ± 0.05a*	44.40 ± 2.62b*	47.60 ± 2.01a*	3514.05 ± 42.39b^	105.88 ± 6.98a^	2	M
	IX	R	0.40 ± 0.06a*	77.51 ± 6.11c*	41.28 ± 3.10a*	2369.70 ± 189.81b*	64.43 ± 3.85a*	2	M
		NR	0.12 ± 0.03a^	25.35 ± 1.20b^	23.10 ± 1.90a^	2578.18 ± 126.80a*	62.57 ± 6.38a*	2	M
KO	V	R	0.51 ± 0.08a*	12.75 ± 2.66a*	35.47 ± 8.80a*	1217.40 ± 269.41a*	44.62 ± 9.38a*	0.1	N
		NR	1.14 ± 0.42b*	19.62 ± 7.68a*	38.56 ± 13.65a*	1935.27 ± 50.44a^	76.17 ± 2.87a^	0.1	N
	IX	R	0.50 ± 0.05a*	33.00 ± 11.17a*	36.98 ± 0.40a*	1083.03 ± 275.37a*	28.88 ± 0.14a*	0.1	N
		NR	0.14 ± 0.02a^	5.83 ± 1.37a^	11.82 ± 5.52a^	1260.95 ± 190.44a*	26.73 ± 6.41a^	0.1	N

The different letters denote significant differences between the particular HNO_3_ extracted metal concentrations in the rhizosphere or non-rhizosphere soils in the same month and different marks denote significant differences between the rhizosphere and non-rhizosphere soils in the same month and in this same sapling sites (*p* < 0.05)

*M* Miasteczko Śląskie, *B* Bukowno, *K* Katowice–Kostuchna, *KO* Kokotek, *V* May, *IX* September, R rhizosphere soil, *NR* non-rhizosphere soil, *PLI* pollution load index, *N* no pollution (PLI < 1), M moderate pollution (1 < PLI < 2), *EH* extremely heavy pollution (3 < PLI)

**Table 6:** The concentration of selected metals (mg kg^−1^) in fractions of the soils extracted with CaCl_2_ (mean values ± SD, *n* = 3) and their potential bioavailability (%)

			Cd		Mn		Zn		Fe		Pb	
M	V	R	16.15 ± 2.75b*	48.00	14.81 ± 2.18a*	12.17	648.60 ± 110.70c*	38.14	0.10 ± 0.03a*	0.00	23.35 ± 2.45b*	1.52
		NR	7.80 ± 0.38d^	73.67	7.71 ± 0.53a^	14.90	389.80 ± 9.30c^	61.06	0.28 ± 0.03a^	0.01	10.75 ± 0.75b^	1.85
	IX	R	15.60 ± 0.50b*	54.73	15.23 ± 0.23a*	19.18	564.25 ± 12.95c*	41.35	0.48 ± 0.02a*	0.01	23.90 ± 1.20b*	1.55
		NR	5.68 ± 0.15c^	92.17	5.28 ± 0.07a^	19.77	183.90 ± 3.14c^	58.00	1.33 ± 0.12a^	0.00	47.89 ± 1.77b^	7.32
B	V	R	1.61 ± 0.03a*	53.42	20.46 ± 0.18b*	58.65	201.11 ± 2.57b*	44.61	29.43 ± 0.49c*	0.78	3.69 ± 0.35a*	1.82
		NR	3.26 ± 0.19c^	43.30	16.03 ± 0.84a^	25.05	237.12 ± 13.72b^	33.83	1.65 ± 0.18ab^	0.04	3.76 ± 1.43a*	1.00
	IX	R	1.01 ± 0.42a*	33.01	8.12 ± 4.04a*	29.10	137.06 ± 54.47b*	28.86	20.35 ± 6.50b*	0.61	3.44 ± 1.25a*	1.67
		NR	2.73 ± 0.09b^	49.82	5.44 ± 0.16a*	12.00	221.80 ± 6.67b*	41.74	0.97 ± 0.07a^	0.03	2.37 ± 0.08a*	0.82
K	V	R	0.40 ± 0.03a*	99.97	65.27 ± 14.28b*	80.28	20.46 ± 0.89a*	53.36	22.43 ± 1.82b*	1.05	2.90 ± 1.86a*	4.42
		NR	0.16 ± 0.07a^	56.51	24.24 ± 8.86b^	55.37	10.11 ± 3.86a^	21.47	3.98 ± 1.41bc^	0.11	3.14 ± 0.69a*	2.99
	IX	R	0.38 ± 0.03a*	85.77	61.03 ± 2.03b*	79.22	20.81 ± 2.76a*	50.89	29.62 ± 7.74b*	1.27	2.20 ± 0.89a*	3.47
		NR	0.11 ± 0.05a^	67.50	9.99 ± 3.32b^	39.66	5.48 ± 2.13a^	24.07	6.18 ± 2.28b^	0.24	3.71 ± 1.09a*	6.08
KO	V	R	0.37 ± 0.12a*	73.76	10.27 ± 1.28a*	83.11	24.78 ± 3.55a*	72.31	34.78 ± 4.68c*	2.95	2.98 ± 0.84a*	6.92
		NR	0.13 ± 0.03a^	13.76	13.85 ± 0.10a*	64.70	34.382.41a*	69.71	6.12 ± 2.38c^	0.32	6.05 ± 4.56ab^	7.90
	IX	R	0.45 ± 0.00a*	89.74	32.03 ± 5.95b*	80.13	25.81 ± 1.89a*	69.77	6.71 ± 1.53a*	0.66	1.00 ± 0.23a*	3.45
		NR	nd	0.00	1.74 ± 0.16a^	31.59	9.23 ± 1.11a^	98.57	16.94 ± 1.74c^	1.38	3.00 ± 0.48a^	11.60

The different letters denote significant differences between the particular CaCl_2_ extracted metal concentrations in the rhizosphere or non-rhizosphere soils in the same month and different marks denote significant differences between the rhizosphere and non-rhizosphere soils in the same month and in this same sapling sites (*p* < 0.05)

*M* Miasteczko Śląskie, *B* Bukowno, *K* Katowice–Kostuchna, *KO* Kokotek, *V* May, *IX* September, *R* rhizosphere soil, *NR* non-rhizosphere soil, *nd* not detected, *potential bioavailability* determined as the percentages of CaCl_2_ extracted metals in relation to the HNO_3_ extracted metals

A particularly high PLI index was found at site M where the levels of heavy metals (HNO_3_ extracted and CaCl_2_ extracted) were statistically significantly higher in the rhizosphere than in the non-rhizosphere soil samples (*p* < 0.05). Generally, the highest levels of Cd, Mn, Zn and Pb (HNO_3_ extracted) were observed at site M in the rhizosphere soil samples. The highest levels (CaCl_2_ extracted) of Cd and Zn were also observed at site M in the rhizosphere soil samples and for Pb in the non-rhizosphere soils samples (Tables 5 and 6).

The following descending order of the potential bioavailability (determined as the percentages of CaCl_2_ extracted metals in relation to the HNO_3_ extracted metals) (Aydinalp and Katkat 2004; Orroño and Alavado 2009) was found among the heavy metals: Cd > Zn > Mn > Pb > Fe in both the rhizosphere and non-rhizosphere soils samples. At site M, despite having the highest heavy metal concentrations in the rhizosphere soil samples, their potential bioavailability was significantly lower than in the non-rhizosphere soil samples. At the other sampling sites, in most cases, the heavy metal potential bioavailability was higher in the rhizosphere soil samples than in the non-rhizosphere soil (Table 6).

### Enzyme activity

**Fig. 2:**
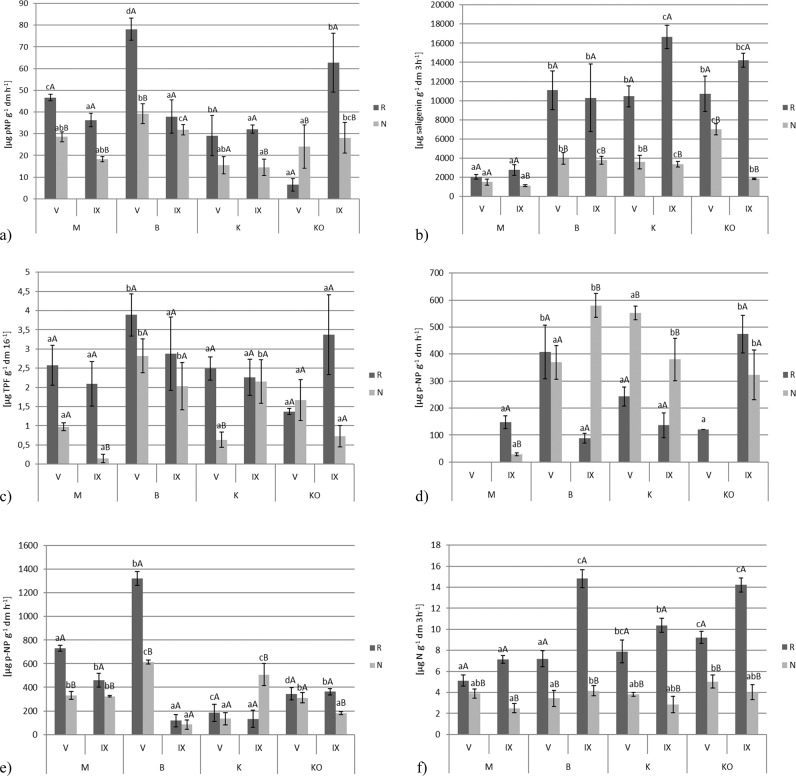
Arylsulphatase (**a**), β-glucosidase (**b**), dehydrogenase (**c**), alkaline phosphatase (**d**), acid phosphatase (**e**), urease (**f**) activities in rhizosphere and non-rhizosphere soil of investigated sites (mean values ± SD, *n* = 3). The different small letters denote significant differences between the particular metal concentrations in the rhizosphere or non-rhizosphere soils in the same month and big letters denote significant differences between the rhizosphere and non-rhizosphere soils in the same month and in this same sampling sites (*p* < 0.05). M Miasteczko Śląskie, B Bukowno, K Katowice–Kostuchna, KO Kokotek, V May, IX September, R rhizosphere soil, NR non-rhizosphere soil

It is clear that the enzyme activity in the V. *myrtillus* rhizosphere soil samples was higher than it was in the non-rhizosphere samples. There was a significant difference (*p* < 0.05) between the enzyme activity in the rhizosphere and non-rhizosphere soils at the same sampling site in the same month. The highest arylsulphatase activity (Fig. 2a) was found in the rhizosphere soil at site B in May and the lowest also in the rhizosphere soil in May at site KO. The β-glucosidase activity (Fig. 2b) was higher at the other sites than at site M in both the rhizosphere and non-rhizosphere soils. The highest activity of this enzyme was observed in May in the rhizosphere soil at site K.

The dehydrogenase (Fig. 2c) levels ranged from 0.15 (μg TPF g^−1^ dm 16^−1^) in the non-rhizosphere soil samples at site M in September to 3.89 (μg TPF g^−1^ dm 16^−1^) in the rhizosphere soil of *V*. *myrtillus* at site B in May. We recorded no alkaline phosphatase (Fig. 2d) activity in May in the rhizosphere and non-rhizosphere soil samples at site M and site KO (only in the non-rhizosphere soil samples). Moreover, the lowest alkaline phosphatase activity was observed in non-rhizosphere soil at M site in September. The highest alkaline phosphatase activity was found in the non-rhizosphere soil at site B.

The highest acid phosphatase activity (Fig. 2e) was recorded in both rhizosphere and non-rhizosphere soil samples at site B in May. At the same time, we observed the lowest activity at the sampling site B in September.

The urease activity (Fig. 2f) was higher in the rhizosphere soil samples collected in September and tended to be highest at sites B and KO while the lowest activity was observed in the non-rhizosphere soil at site M. We found that the enzyme activity did not show any clear seasonal patterns.

Based on the soil enzyme activity, the highest values of the indexes TEI and *M*_*w*_ (Table 7) were found in the *V. myrtillus* rhizosphere soil samples. The highest value of TEI was found at site B in rhizosphere soil in May and the lowest in the non-rhizosphere soil from site M in September. In the case of the *M*_*w*_ index, the highest value was observed in the rhizosphere soil at site KO, while the lowest was in the non-rhizosphere soil at site M.

**Table 7:** Chosen indexes and indices of soil samples

			TEI	*M*_*w*_	ACR					
					AS	βG	Deh	AlP	AP	Ure
M	V	R	5.68a*	130.99a*	620.09	−80.93	87.84	0	111.56	−93.60
		NR	3.03a^	38.04a^	18.91	−78.58	−41.62	0	6.21	−91.06
	IX	R	5.45a*	106.48a*	−4.18	−80.55	−37.86	−68.98	26.72	−94.21
		NR	2.14a^	23.38a^	−35.07	−38.40	−79.00	−91.02	77.47	−92.81
B	V	R	12.23b*	857.13c*	1105.85	3.56	183.75	239.34	282.79	−91.00
		NR	6.85b^	234.52c^	62.98	−43.30	68.80	0	96.88	−92.12
	IX	R	7.06a*	135.35a*	−39.61	−27.69	−14.65	−81.49	−67.70	−87.98
		NR	5.81c^	95.29bc^	13.25	106.10	178.29	79.77	−53.97	−88.05
K	V	R	6.40a*	167.46ab*	349.08	−2.44	81.67	101.80	−46.52	−90.14
		NR	4.56a^	124.48b*	−35.14	−48.59	−62.04	0	−56.82	−91.25
	IX	R	7.13a*	129.65a*	−48.79	17.05	−32.98	−71.33	−63.45	−91.57
		NR	5.35b*	151.04c*	−48.69	83.96	194.75	17.68	177.22	−91.78
KO	V	R	5.31a*	219.9b*	0	0	0	0	0	0
		NR	4.21a*	85.11ab^	0	0	0	0	0	0
	IX	R	10.82b*	577.58b*	0	0	0	0	0	0
		NR	3.92ab^	44.79ab^	0	0	0	0	0	0

The different letters denote significant differences between particular indexes (TEI and M_*w*_) in the rhizosphere or non-rhizosphere soils in the same month and different marks denote significant differences between the rhizosphere and non-rhizosphere soils in the same month and in this same sapling sites (*p* < 0.05)

*M* Miasteczko Śląskie, *B* Bukowno, *K* Katowice–Kostuchna, *KO* Kokotek, *V* May, *IX* September, *R* rhizosphere soil, *NR* non-rhizosphere soil, *TEI* total enzyme activity index, *M*_*w*_ potential biochemical soil fertility index, *ACR* enzyme activity change ratio, *AS* arylsulphatase, *βG* β-glucosidase, *Deh* dehydrogenase, *AlP* alkaline phosphatase, *AP* acid phosphatase, *Ure* urease

### The effect of heavy metal pollution and other soil properties on the soil enzyme activity

**Fig. 3:**
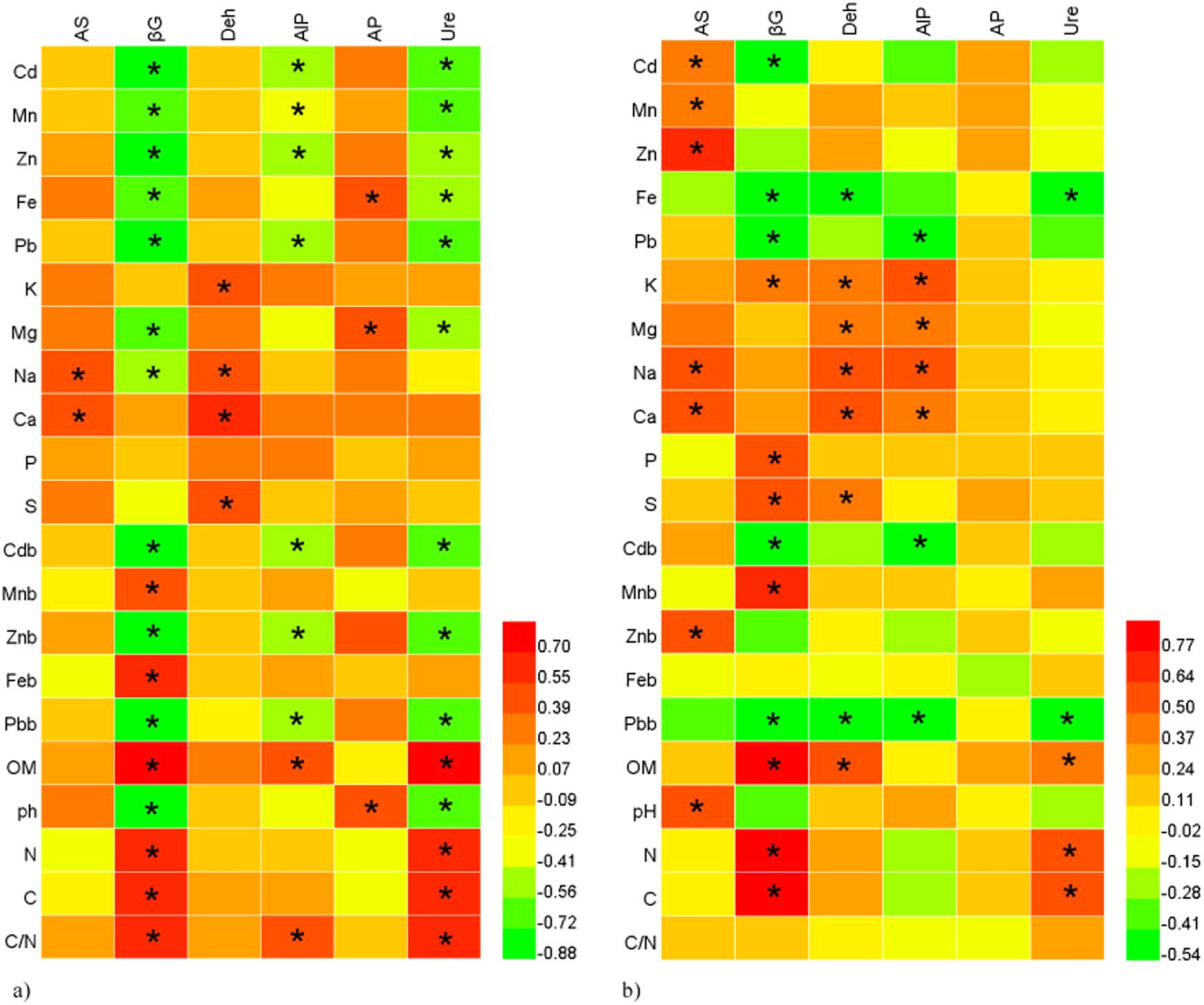
Heat map of the correlation between soil enzyme activities and properties in rhizosphere (**a**) and non-rhizosphere (**b**) soils. Strong positive correlation (red), weak correlation (yellow), strong negative correlation (green); *significant correlation (*p* < 0.05). AS arylsulphatase, βG β-glucosidase, Deh dehydrogenase, AlP alkaline phosphatase, AP acid phosphatase, Ure urease, OM organic matter, b potentially bioavailable elements

A more severe impact of heavy metals and soil properties on soil enzyme activity was observed in the *V. myrtillus* rhizosphere soil samples than in the non-rhizosphere soil samples at the same site (Fig. 3a, b). Generally, the activity of the soil enzymes, especially β-glucosidase and urease, decreased with increasing heavy metal concentrations for both the CaCl_2_ and HNO_3_ extracted values. A positive correlation coefficient was also obtained between β-glucosidase and urease and soil properties such as the organic matter content and the *C* and *N* concentrations. There were highly significant correlations between the activity of all of the investigated soil enzymes and the macronutrient levels in both the rhizosphere and non-rhizosphere soils (Fig. 3a, b).

**Fig. 4:**
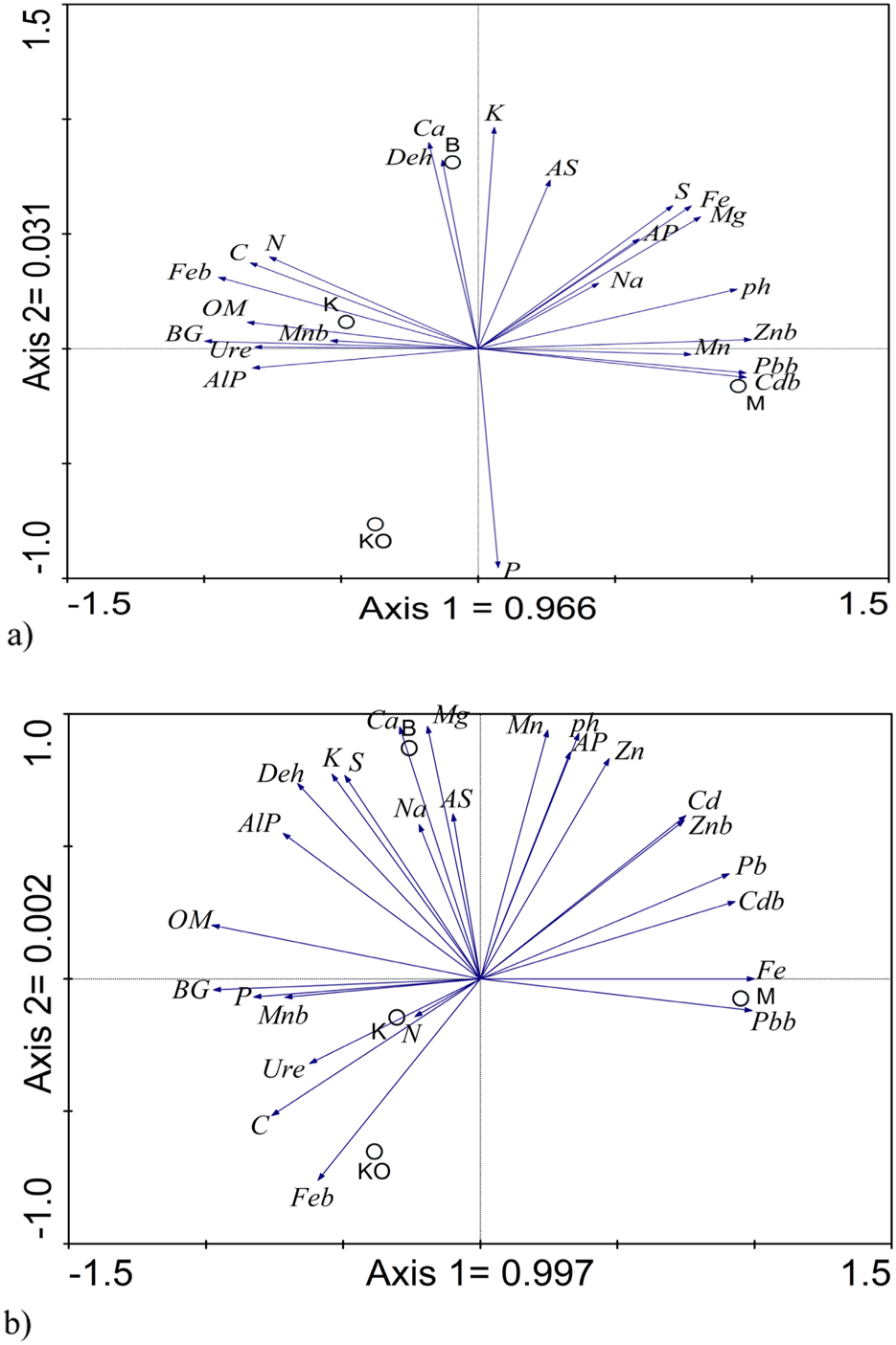
Principal Component Analysis (PCA) biplot of sampling sites and heavy metal concentrations and enzyme activities and biochemical parameters in the rhizosphere (**a**) and non-rhizosphere (**b**) soil samples. M Miasteczko Śląskie, B Bukowno, K Katowice–Kostuchna, KO Kokotek, AS arylsulphatase, BG β-glucosidase, Deh dehydrogenase, AlP alkaline phosphatase, AP acid phosphatase, Ure urease, OM organic matter, Xb potentially bioavailable elements

A PCA analysis of the rhizosphere soils (Fig. 4a) showed that the factors that correlated with the first axis explained 96.6% of the variability, thus indicating negative correlations between the β-glucosidase, urease and alkaline phosphatase activity and the pH value and the Zn, Pb, Cd content (CaCl_2_ extracted), but a positive correlation between these enzymes and the *C*, *N*, OM content. In turn, for the non-rhizosphere soils (Fig. 4b), the first axis explained 99.7% of the variability. The factors for which there was a positive correlation were the β-glucosidase activity and *P* content, urease activity and *C* content and acid phosphatase and pH value. Negative correlations were found between the alkaline phosphatase and dehydrogenase activity and the Cd, Znb, Pb and Cdb content.

The ACR (enzyme activity change ratio) varied from −94.21 to 1105.85 for the *V. myrtillus* rhizosphere soil and from −91.78 to 194.75 for the non-rhizosphere soil (Table 7).

### Biological indices

A total of 20 179 microarthropods from nine different taxa were extracted from the soil samples (Table 8). Mites were by far the most abundant taxa in the samples (Table 8). The highest number of microarthropods (2486) were collected in the rhizosphere soil samples at site KO. The highest value of the QBS and FEMI indices were found in the rhizosphere soil at the control site KO and the lowest in the non-rhizosphere soil samples at contaminated site M (Table 8).

**Table 8:** Abundance of microarthropod groups and QBS and *F*_*EMI*_ indices at the studied sites

			Acari	Collembola	Chilopoda	Pseudoscorpionida	Aranea	Diptera larvae	Diptera adults	Hymenoptera	Diplopoda	QBS	*F*_*EMI*_
M	V	R	351	664	0	10	8	14	19	31	1	72	0.010
		NR	81	121	0	0	0	90	2	37	0	37	0.006
	IX	R	266	416	0	8	3	21	32	6	0	62	0.006
		NR	136	102	0	1	0	16	4	2	0	62	0.002
B	V	R	1360	252	3	71	13	13	10	18	0	72	0.022
		NR	1026	277	0	46	26	18	7	9	1	72	0.011
	IX	R	549	102	0	7	9	9	1	3	15	72	0.030
		NR	577	178	0	31	0	4	2	2	3	72	0.014
K	V	R	1413	573	1	1	2	8	2	8	3	82	0.017
		NR	587	242	4	1	2	58	5	4	1	82	0.016
	IX	R	716	257	4	2	10	20	12	0	5	82	0.015
		NR	699	261	4	1	7	12	7	2	4	82	0.015
KO	V	R	1635	736	1	5	22	36	12	38	1	102	0.045
		NR	1679	702	7	10	18	12	4	21	2	82	0.030
	IX	R	1856	367	5	8	35	20	12	58	0	72	0.029
		NR	747	84	0	9	0	11	3	0	1	62	0.007

*M* Miasteczko Śląskie, *B* Bukowno, *K* Katowice–Kostuchna, *KO* Kokotek, *V* May, *IX* September, *R* rhizosphere soil, *NR* non-rhizosphere soil, *TEI*, *QBS* soil biological quality index, *FEMI* abundance-based fauna index

## Discussion

### Assessment of heavy metal pollution

Very high total concentrations of the studied heavy metals were observed in the soils from Miasteczko Śląskie (site M). The concentrations of Cd, Zn and Pb exceeded the permissible levels according to the Regulations of the Polish Minister of the Environment (2002). Under this regulation, the maximum allowable concentrations of these metals in the soil should not exceed 3 mg kg^−1^, 300 mg kg^−1^ and 100 mg kg^−1^, respectively. Excessive concentrations of Cd, Zn and Pb were also found at Bukowno (site B). These results were like our previous studies that had been carried out in the same or similar areas (e.g., Kandziora-Ciupa et al. 2017).

At Miasteczko Śląskie, the rhizosphere soils accumulated more heavy metals than the non-rhizosphere soils, while at the other sampling sites the heavy metal content was lower in the rhizosphere than in the non-rhizosphere soils in most cases.

In this study, the pollution load index was also determined, which was particularly high at site M. We agree with Yang et al. (2016) that PLI is a simple and useful means to assess the overall level of heavy metal pollution between different sampling sites.

Many studies have emphasized that the total concentration of heavy metals in soils provides little information on their mobility, bioavailability and, hence, their potential toxicity (Feng et al. 2005; Boussen et al. 2013; Wójcik et al. 2014). It has been widely accepted that heavy metal availability has a major influence on the toxic effects in biological systems (Olaniran et al. 2013; Yang et al. 2017). For the extraction of heavy metals, a 0.01 M CaCl_2_ solution has been proposed as the most preferred extraction medium solution (Menzies et al. 2007; Boussen et al. 2013). The following descending order of bioavailability was found for the heavy metals that were analyzed: Cd > Zn > Mn > Pb > Fe in both the rhizosphere and non-rhizosphere soils samples. Similar results were found by Szarek-Łukaszewska and Niklińska (2002) in Zn–Pb ore tailings or their immediate vicinity and by Wójcik et al. (2014) in Zn–Pb waste deposits in southern Poland.

At site M, the bioavailability of the heavy metals in the rhizosphere soils was significantly lower than in the non-rhizosphere soils samples, and the reason was attributed to an increase in pH in the rhizosphere soil (Wang et al. 2002). Among soil properties, soil pH had the greatest impact on the desorption and bioavailability of heavy metals, because of its strong effects on solubility and speciation of heavy metals both in the soil as a whole and particularly in the soil solution (Müehlbachová et al. 2005; Chen et al. 2013). At high pH, metals tend to form insoluble metal mineral phosphates and carbonates, whereas at low pH they tend to be found as free ionic species or as soluble organometals and are more bioavailable (Olaniran et al. 2013). At the other sampling sites, in most cases, heavy metal bioavailability was higher in the rhizosphere soils samples. Yang et al. (2017) detected that the availability of Pb was significantly higher in bulk soil than that in rhizosphere soil at medium and high pollution levels. Low molecular weight organic acids (e.g., oxalate), which are released by plant roots can form complexes with heavy metals, which can immobilize and reduce their availability in rhizosphere soil (Wang et al. 2002; Luo et al. 2017).

### Soil enzyme activity

#### Rhizosphere effects on enzyme activity

In general, all of the soil enzyme activity that was measured in this study exhibited higher values in the rhizosphere soils than in the non-rhizosphere soil. The higher enzyme activity of the rhizosphere may be due to the physiological activity of the roots, which produce and release large amounts of enzymes under the influence of heavy metals or *via* the lysis of root cells. Moreover, it may also depend on the physiological metabolic activities of the diverse microbial populations, which produce specific soil enzymes (Gianfreda 2015; Xiao et al. 2017; Yang et al. 2017).

#### Effects of the soil properties and macronutrient content on the enzyme activity

In this study, the physicochemical properties of the rhizosphere soils such as the levels of OM, C, N, K, Mg, Na, Ca, P and S were significantly higher than those of the non-rhizosphere soils. According to Wang et al. (2009), this may be the result of the exudates and metabolites that are released by the roots and microbial metabolites in rhizosphere soil. Garcia et al. (2005) found that in all six investigated plant species, there was an increase in the organic matter content in the rhizosphere.

A factor that may largely determine soil enzyme activity is the organic matter content, which correlates with several biochemical functions of the soil enzymes (Nannipieri et al. 2002; Wahsha et al. 2017). The organic matter content provides a better soil environmental condition for stabilizing and protecting enzymes. A higher organic carbon content supports a larger microbial biomass, which leads to an increase in the enzymatic activity (Bartkowiak et al. 2017). Many authors have found positive correlations between soil enzyme activity and organic matter content (e.g., Tan et al. 2014; Gucwa-Przepióra et al. 2016). In the present study, we found a statistically significant correlation between the activity of β-glucosidase, alkaline phosphatase, and urease in the rhizosphere soils, along with the dehydrogenase and organic matter content in the non-rhizosphere soils. This supports the findings of Patel and Patra (2014), who found that the activity of acid and alkaline phosphatase and dehydrogenase in tannery sludge that was rich in heavy metals were positively correlated with organic matter.

Soil pH has been identified as one of the key abiotic environmental factors shape soil enzyme activity in soils (Turner 2010; Pan et al. 2018). Effron et al. (2004) reported that the enzyme activity was sensitive to pH changes and that different enzymes can respond differently to the same pH. In this study, the urease and beta-glucosidase activity levels were negatively correlated with the soil pH values, while by contrast, the acid phosphatase and arylsulphatase levels increased with higher soil pH values. In rhizosphere soils, changes in soil pH values have a stronger influence on the enzyme activity than in non-rhizosphere soil. Previous studies have reported that urease, β-glucosidase, and acid phosphatase are significantly affected by changes in soil pH (Acosta-Martinez and Tabatabai 2000; Hinojosa et al. 2004).

Analysis of the data showed that there were highly significant correlations between all of soil enzyme activity and macronutrient contents in both the rhizosphere and non-rhizosphere soils that were investigated. According to Chróst (1991), microorganisms control their enzyme production in response to nutrient availability. The strong dependency between soil enzyme activity and different soil properties confirms that soil enzyme activity provides a meaningful integrative measure of the soil physicochemical properties and biological soil fertility, which, thus, may play a role in monitoring soil biological quality (Aşkın, Kızılkaya 2006; Tan et al. 2014).

In contrast, Wyszkowska and Wyszkowski (2003) proposed a potential biochemical soil fertility index (*M*_*w*_), which could be more efficient in making predictions about soil fertility than the activity of a single soil enzyme. In the present study, we found that the *M*_*w*_ was higher in the rhizosphere soil; however, the *M*_*w*_ index did not correlate with. the studied soil properties.

#### Effects of heavy metals on the enzyme activity

Analysis of many different enzyme activities can provide a better picture of the status of soil processes and functioning (Acosta-Martinez et al. 2003), therefore, in this study, we examined the activity of six soil enzymes.

The effects of heavy metals on the enzyme activity are complex. The response of different enzymes to the same metal can vary greatly and the same enzyme may respond differently to different metals (He et al. 2003; Li et al. 2009).

The effects of heavy metals on soil enzyme activity have been reported in many previous studies, but they were mostly concentrated on the impact of the total concentrations of heavy metals on these enzymes. However, as was previously mentioned, biological systems and soil quality are more dependent on the bioavailable heavy metal concentration (Hinojosa et al. 2004). Therefore, we focused on the effect of the potentially bioavailable heavy metal fraction on enzyme activity. In this study, the various soil metals showed effects on soil enzyme activity and these effects differed between the rhizosphere and non-rhizosphere soils.

β-glucosidase is a useful indicator of soil quality and its activity may indicate changes in the level of organic carbon earlier than measurements using other methods (Das and Varma 2011). This enzyme plays an important role in the degradation of the organic C compounds in the soil and are important energy sources for microorganism (Acosta-Martinez and Tabatabai 2000; Narendrula-Kotha and Nkongolo 2017). Turner et al. (2002) and Hinojosa et al. (2004) found that β-glucosidase is a decisive indicator of soil contamination by toxic metals. In our study, these findings were supported because we found the lowest β-glucosidase activity at the site with the highest Cd, Zn and Pb concentrations. These metals strongly inhibited the β-glucosidase activity in both the rhizosphere and non-rhizosphere soils. In contrast, Narendula-Kotha and Nkongolo (2017) reported a higher β-glucosidase activity in metal-contaminated sites. Similarly, in our work, we found a positive correlation between the Mn and Fe and β-glucosidase activity.

Urease catalyzes the hydrolysis of urea in soil, which induces the formation of carbon dioxide and ammonia (Baćmaga et al. 2015). In the present study, the correlation coefficients confirmed that urease activity was negatively correlated to Cd, Zn, Pb in the rhizosphere soils and to Pb in the non-rhizosphere soils. Similar results were obtained by Angelovičová et al. (2014) who found that Pb and Zn decreased the urease activity levels in soils. According to Gao (2010), urease appears to be more sensitive to pollution stress than phosphatases.

Phosphatase plays an important role in transforming organic phosphorus into an inorganic form that is suitable for plant uptake (Cang et al. 2009; Angelovičová et al. 2014). We observed an inhibitory effect of Cd, Pb and Zn on the alkaline phosphatase activity. Also, Pattnaik and Equeenuddin (2016) found that alkaline phosphatase was negatively correlated with the examined metals except for Pb. Wahsha et al. (2017) reported negative correlations between Fe, Pb, Zn and Cu and alkaline phosphatase.

Dehydrogenase plays a significant role in soil through the biological oxidation of soil carbon and transfers the hydrogen ion from organic substrates to inorganic substances (Zhang et al. 2008). Dehydrogenase is most sensitive to heavy metal pollution (Khan et al. 2007). In the present study, we only found a negative correlation between Pb and dehydrogenase activity in the non-rhizosphere soils. Similar results were obtained by Pan and Yu (2011), who found that the dehydrogenase activity decreased after significantly after two- and fou-week Pb500 treatments. Wyszkowska et al. (2006) observed that the application of 50 mg/kg Pb significantly reduced the dehydrogenase activity in soil.

Arylsulphatase plays is an indicator of sulphur mineralization in soil and is an important part in the cycling of this element (Lipińska et al. 2014). Its activity depends on several factors including heavy metals (Kang and Freeman 1999). However, we only found a positive correlation between this enzyme and the Zn concentration in the non-rhizosphere soils.

Additionally, in order to determine the relative toxicity of heavy metals to enzyme activity in the studied soils, we determined the enzyme activity change ratio (ACR). A positive ACR denotes that enzyme activity is enhanced and a negative ACR denotes that enzyme activity is reduced in the presence of heavy metals (Xian et al. 2015). In our study, a negative ACR primarily concerned urease and to a lesser extent the β-glucosidase activity. Gucwa-Przepióra et al. (2016) found a negative ACR in soil, which confirmed the inhibition of soil enzyme activity at a site that had been affected by smelting activity. Xian et al. (2015) indicated that for Cd- and As-polluted soils, the ACRs of the enzymes in the soils did not exhibit a consistent rise and fall pattern.

In order to assess the total level of soil enzyme activity, we also calculated the integrated total enzyme activity index (TEI). The TEI was higher in the rhizosphere soil samples from all of the investigated sites, but we did not observe any differences between the sampling sites or correlations between the TEI and the studied soil properties. In contrast to our study, Tan et al. (2014) found positive correlations between the TEI and soil OM, total N and negative correlations between the soil pH values. Additionally, Fang et al. (2017) reported a positive correlation between the TEI and electrical conductivity.

In our study, individual enzyme activity had a stronger correlation with the soil physicochemical properties than the two indices: TEI and *M*_*w*_ that were calculated.

### Biological indices

This study was conducted at sites with different degrees of soil contamination and each of the investigated sites is described by a characteristic structure of soil microarthropod community. The soils from Miasteczko Śląskie (M) had the lowest number of microarthropod taxa as well as a low QBS and FEMI, which can be explained by the remarkably high concentrations of heavy metals at that site. Moreover, Santorufo et al. (2012) found a lower QBS in heavy-metal contaminated soils. Our results confirmed that heavy metals contamination affects QBS-ar values. In most cases the compared to the values obtained for human degraded soils e.g., from Poland, the UK, Sweden, and Italy where the other specialists obtained values of this biological indicator between 40 and 70 (Menta et al. 2018). This indicates that QBS and FEMI are quite sensitive to the habitat changes that are caused by the anthropogenic impact on soil. Similar results were found by Madej et al. (2011) and Menta et al. (2018). Pollution not only affects mesofauna by toxicity, but it can also cause some indirect effects through changes in the quantity and quality of the soil organic matter content and the associated microbial communities (Khalil et al. 2009). Many authors (Gwiazdowicz et al. 2006; Cui et al. 2016; Manu et al. 2017) have indicated a positive correlation between the number of microarthropods and the content of organic matter, TOC and C/N in the soil. The higher content of the organic matter and the lower bioavailability of the heavy metals resulting from the presence of plants influence rhizosphere soil in a way that makes the living conditions for microarthropod better. That means that rhizosphere soil has a higher biological quality compared with non-rhizosphere soil. A higher number of microarthropods, as well as higher values of the QBS and FEMI indices, were found in the rhizosphere soil regardless on the degree of contamination on specific sites.

The soil fauna is a good tool to assess soil biological quality due to its complex nature. However, in this study, the FEMI index seems to be a better indicator of the differences in soil quality both at the sites and the respective soil layers (i.e., the rhizosphere and non-rhizosphere soil). Because the FEMI considers both the presence and abundance of individual microarthropod taxa, it can assess soil quality more realistically than the QBS, which was confirmed in the results that were presented by Yan et al. (2012).

## Conclusions

Based on the heavy metal content levels that were determined, their availability and their influence on soil enzyme activity and microarthropod communities in the *Vaccinium myrtillus* L. rhizosphere soil and the non-rhizosphere soil from sites with different degrees of pollution, the major findings of this study are as follows:

(1) The physicochemical and biological properties of the *V. myrtillus* rhizosphere soil was significantly different from those of the non-rhizosphere soil. Our results showed that the heavy metals had various patterns of mobility between the rhizosphere and non-rhizosphere soils at different polluted sites. The heavy metal bioavailability was generally higher in the rhizosphere soil samples except for the most polluted site—Miasteczko Śląskie. (2) In the *V. myrtillus* rhizosphere soil samples, enzyme activity was generally higher than in the non-rhizosphere soil. β-glucosidase and urease were strongly impacted by the organic matter content, the C and N levels and pH values. Moreover, these enzymes were most sensitive to Cd, Zn and Pb, which makes them good indicators for detecting the impact of heavy metal pollution in forest ecosystems. (3) The *Vaccinium myrtillus* L. rhizosphere soil had stronger correlation coefficient values between the measured parameters than the non-rhizosphere soil, which suggests that rhizosphere soil is more sensitive and could be used in the monitoring and assessment of forest ecosystems. (4) The QBS and FEMI methods, which are based on microarthropod communities, is a sensitive tool that can be used to assess the degree of soil degradation. Because of the conjunction between these methods and the soil physicochemical properties as well as level of contamination and other biological parameters such as soil enzyme activity, a proper assessment of soil quality is possible.

There is still a lack of knowledge on the impact of heavy metals on changes in the activity of soil enzymes and microarthropod communities in the rhizosphere of selected species growing in field conditions. The results that were obtained in our study cannot be interpreted in an unambiguous way and they only provide an indication of the effect of heavy metal contamination on the rhizosphere. However, this type of research, by identifying sensitive indicators, may help to improve the monitoring and assessment of forest ecosystems.

## Data Availability

The data presented in this study are available on request from the corresponding author. The data are not publicly available due to privacy restrictions.

## Abbreviations

AS: arylsulphatase
βG: β-glucosidase
Deh: dehydrogenase
AlP: alkaline phosphatase
AP: acid phosphatase
Ure: urease
PLI: pollution load index
TEI: total enzyme activity index
*M*_*w*_: potential biochemical soil fertility index
ACR: enzyme activity change ratio
QBS: soil biological quality index
FEMI: abundance-based fauna index
